# Positive-electrode properties and crystal structures of Mg-rich transition metal oxides for magnesium rechargeable batteries

**DOI:** 10.1038/s41598-022-23022-1

**Published:** 2022-10-27

**Authors:** Naoto Kitamura, Yoichiro Konishi, Wenli Ma, Naoya Ishida, Toshihiko Mandai, Chiaki Ishibashi, Yasushi Idemoto

**Affiliations:** 1 Department of Pure and Applied Chemistry, Faculty of Science and Technology, Tokyo University of Science, 2641 Yamazaki, Noda, Chiba 278-8510 Japan; 2 Research Group for Advanced Energy Conversion, Research Institute for Science and Technology, Tokyo University of Science, 2641 Yamazaki, Noda, Chiba 278-8510 Japan; 3 Center for Green Research On Energy and Environmental Materials, Center for Advanced Battery Collaboration, National Institute for Materials Science, Tsukuba, Ibaraki 305-0044 Japan

**Keywords:** Energy storage, Condensed-matter physics, Electrochemistry, Inorganic chemistry

## Abstract

In this work, we focus on Mg–Fe–O and Mg–Ni–O with Mg-rich compositions as positive-electrode materials for magnesium rechargeable batteries, and prepare them by a thermal decomposition of precipitates obtained by a solution method. It is indicated from X-ray diffraction patterns that the Mg–Fe–O and Mg–Ni–O samples have the spinel and rocksalt structures, respectively. X-ray absorption near edge structures indicate that Fe and Ni are trivalent and divalent, respectively, in the Mg-rich oxides. From charge/discharge cycle test, it is demonstrated that the Mg–Fe–O shows higher discharge capacity than the other and then has good cycle performance while keeping a discharge capacity over 100 mA h g^–1^. To gain deeper understanding on a relationship between the electrode properties and the crystal structure of the Mg–Fe–O, the crystal structure is investigated by a Rietveld refinement using a synchrotron X-ray diffraction profile and an analysis on total correlation functions. It is indicated from these studies that a vacant octahedral site in the spinel structure is partially occupied by the excess Mg in the synthesized sample. This structural feature might result in a stable charge/discharge cycle performance of the Mg-rich Mg–Fe–O.

## Introduction

As is well known, rechargeable batteries like lithium-ion batteries have been used as energy sources of portable electronic devices in the last few decades. In recent years, however, the batteries are expected to be applied for large stationary systems to enhance usage efficiencies of renewable energies and thus reduce environmental load. To realize such a system, it is considered as a key issue to develop a novel battery with higher energy density than commercialized lithium-ion batteries. As post lithium ion batteries, magnesium rechargeable batteries (MRB) have drawn much attention since Aurbach et al. proposed the prototype batteries which used a sulfide and a Mg metal as the positive and negative electrode materials, respectively^1^. One of the most important advantages is a fact that a higher energy density can be achieved theoretically in MRB compared with the lithium ion batteries due to higher valence of Mg ion than Li ion. On the other hand, many researchers have suffered from inferior Mg ion diffusion in electrode materials of MRB because the higher valence of Mg ion (Mg^2+^) induces strong electrostatic interaction between Mg^2+^ and anions. To overcome this problem, transition-metal oxides with various crystal structures have been investigated actively in the last decade, and Mg*M*_2_O_4_ with a spinel structure^2–20^, Mg*M*O_2_ with a disordered rocksalt structure^21^, ZnMnO_3_ with a deficient spinel structure^22,23^, magnesiated Li_x_*M*_1–y_O_2_ with a layered structure (*M*: transition metals)^24–26^ were reported as promising candidates for the positive-electrode materials of MRB, although the electrode properties are still insufficient for a commercial use unfortunately. To establish a guideline for development of novel positive-electrode materials with better electrochemical properties, most of the previous works paid special attention on the crystal structures since diffusion mechanisms of Mg^2+^ in the electrode materials must be related to their atomic configurations. In addition, some studies focused on a Mg/*M* ratio and investigated effects of the ratio on electrochemical properties and crystal structures. For example, Mg(Co, Mn)_2_O_4_ nanoparticle exhibited higher discharge capacity when the material had slight excess amount of Mg, i.e., Mg_1.04_Co_1.46_Mn_0.6_O_4_, and a spinel-type Mg–V–O material with high Mg/V ratio, Mg(Mg_0.5_V_1.5–x_Ni_x_)O_4_, showed relatively good cycle performances, as reported previously^15,20^. At the moment, however, there are a few works on positive-electrode materials with Mg-rich composition.

From such background, we tried to prepare two kinds of magnesium transition-metal oxides with Mg-rich compositions. One of the oxides is a Mg–Fe–O-based material with a spinel structure. This is because Fe-containing oxides with a spinel structure can suppress electrolyte decomposition significantly during a charging process and thus are expected to show superior cycle performance^9,16^. To obtain the spinel oxide with Mg-rich composition, we focused on a thermal decomposition method of a layered double hydroxide (LDH) as a synthetic process, taking previous works on Mg–Fe LDH with high Mg/Fe ratios into account^27,28^. The other oxides with a Mg-rich composition is a Mg–Ni–O with a rocksalt structure since a previous work had an investigation only on the rocksalt oxide with Mg-poor composition^21^. These obtained products with Mg-rich compositions were investigated by charge/discharge cycle tests and atomic-configuration analyses using synchrotron X-ray diffraction patterns.

## Results and discussion

**Figure 1:**
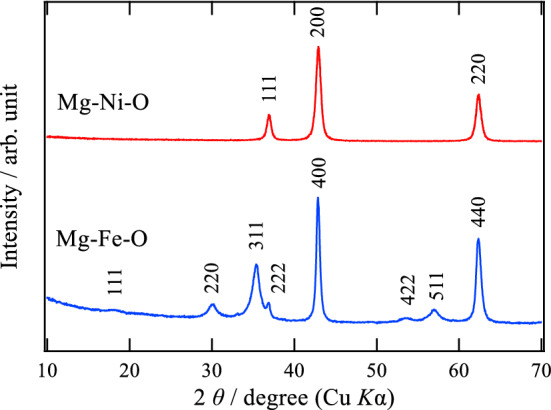
X-ray diffraction patterns of Mg–Fe–O and Mg–Ni–O after the thermal decomposition.

**Figure 2:**
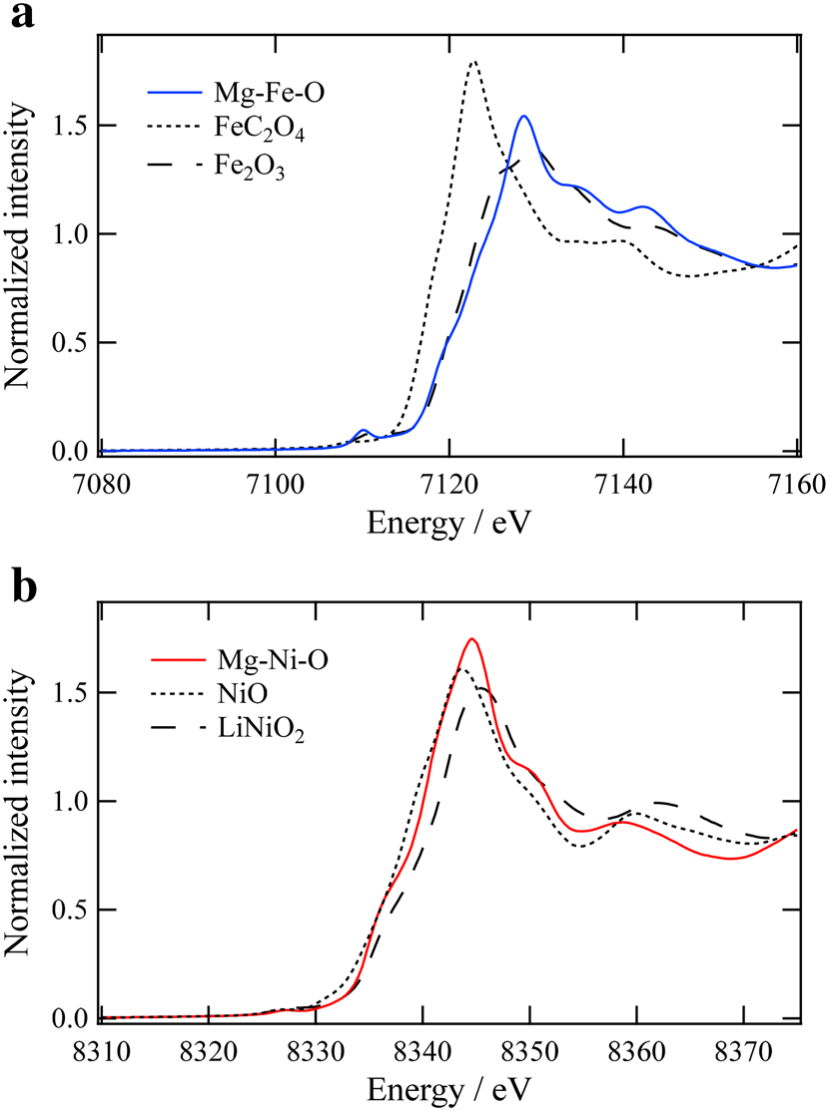
XANES spectra at (**a**) Fe *K*-edge of Mg–Fe–O and (**b**) Ni *K*-edge of Mg–Ni–O with reference materials.

Figure 1 shows X-ray diffraction patterns (Cu *K*_α_) of the Mg–Fe–O and Mg–Ni–O after the final firing process. As can be seen in this figure, these samples have different crystal structures apparently. Regarding the Mg–Fe–O sample, the Bragg peaks can be attributed to the spinel structure although the detailed structure is discussed below. On the other hand, the Mg–Ni–O specimen has a single phase of the rocksalt structure. Table 1 lists metal composition ratios of these oxides estimated by an inductively coupled plasma atomic emission spectroscopy (ICP-AES). It is found that the analytical compositions are essentially equal to the nominal values. This result suggests that the Mg-rich samples can be successfully synthesized by means of the preparation method applied in this work. For these samples, X-ray absorption near edge structure (XANES) spectra of the transition metals were also measured to evaluate the valences, and the results are shown in Fig. 2. It is demonstrated from the spectra that Fe in the Mg–Fe–O is trivalent and Ni in the Mg–Ni–O is divalent. Taking these results into account, the Mg–Ni–O sample can be expressed as Mg_0.693_Ni_0.307_O with the rocksalt structure, which is a solid solution of rocksalt-type MgO and NiO with divalent cations. On the other hand, the Mg–Fe–O has the spinel (AB_2_O_4_)-based structure, which can be regarded as a metal-deficient rocksalt structure due to the higher metal valence of Fe than divalent. Since the oxygen content in the sample can be assumed to be 4, a formula of the Mg-rich Mg–Fe–O can be described as Mg_2.446_Fe_1.036_O_4_ on the basis of the electroneutrality condition, 2[Mg^2+^] + 3[Fe^3+^] = 2[O^2–^], and the analytical composition ratio (Table 1).

**Table 1:** Metal composition ratios of Mg–*M*–O (*M* = Fe or Ni) after the decomposition of the precipitates.

Sample	Mg	Fe	Ni	Mg/*M*
Mg–Fe–O	0.702(1)	0.298(1)	–	2.36
Mg–Ni–O	0.693(2)	–	0.307(1)	2.26

**Figure 3:**
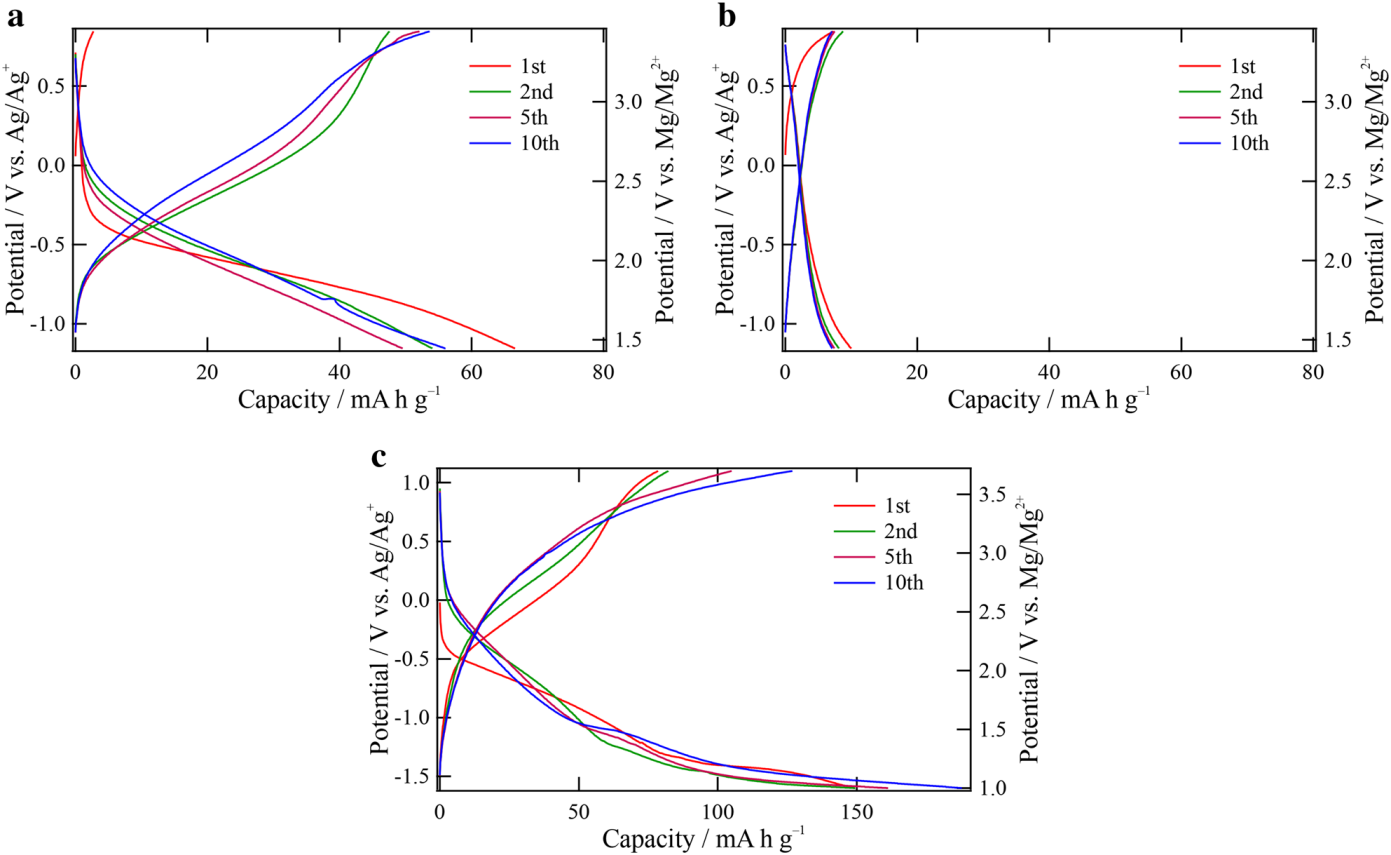
Charge/discharge curves of (**a**) Mg–Fe–O and (**b**) Mg–Ni–O with a cut-off potential of − 1.155 ~ 0.845 V vs. Ag/Ag^+^ (1.445 ~ 3.445 V vs. Mg/Mg^2+^). (**c**) Charge/discharge curves of Mg–Fe–O with a cut-off potential of − 1.6 ~ 1.1 V vs. Ag/Ag^+^ (1.0 ~ 3.7 V vs. Mg/Mg^2+^) are also presented. All the measurements were carried out at 90 °C with the current density of 5 mA g^–1^.

Figure 3a and b show charge/discharge curves of the Mg–Fe–O and Mg–Ni–O samples with a cut-off potential of − 1.155 ~ 0.845 V versus Ag/Ag^+^. In both the samples, the first charge capacities are very low (less than 10 mA h g^–1^), suggesting that the transition metals cannot be oxidized within the potential range and thus Mg cannot be removed from the crystals. As for the Mg–Fe–O, the first discharge capacity was around 65 mA h g^–1^, and this capacity can be considered to be induced by Mg^2+^ insertion into the vacant site (the 16*c* site) of the spinel structure (Fig. S1)^4^. It is also seen in the figure that the cycle performance of the Mg–Fe–O with the spinel structure is rather good, and this result is in accordance with a previous work on Mg(Mn, Fe)_2_O_4_^9^: i.e., MgFe_2_O_4_ exhibited the highest capacity-retention rate among Mg(Mn, Fe)_2_O_4_. Moreover, it is supposed that the Mg-rich sample can deliver capacities comparable to MgFe_2_O_4_ even though the cut-off potential applied here is higher for the discharge than the literature^9^. From the result, it can be considered that the high Mg/Fe ratio can improve the positive-electrode properties. On the other hand, the capacity of the Mg–Ni–O is very low even after the first discharge process. This means that the Mg-rich composition does not have a positive influence on the electrode properties in the case of rocksalt-type materials.

To investigate positive-electrode properties of the Mg–Fe–O with the Mg-rich composition deeply, we made the cut-off potential wider, and give the experimental result in Fig. 3c. This cycle test was started from a discharge process since Mg cannot be extracted from the crystal as mentioned above. Both the discharge and charge capacities are enhanced significantly by changing the cut-off potential and are beyond 100 mA h g^–1^ after several cycles, although the charge capacities are smaller than the discharge capacities, indicating unexpected reactions at the low potential region. The metal composition of the electrode after the discharge was also investigated by ICP-AES to evaluate the Mg insertion amount. As a result, the Mg insertion amount during the discharge was estimated as 0.475, corresponds to 140 mA h g^–1^. This analysis supports that the Mg-rich sample can deliver higher capacity than 100 mA h g^–1^, although the estimated capacity is deviated from the electrochemically-obtained discharge capacity due to unexpected reaction in this experimental condition. From these results, the Mg–Fe–O-based spinel oxide with the Mg-rich composition can be regarded as one of the promising candidates of a positive-electrode material of MRB.

**Figure 4:**
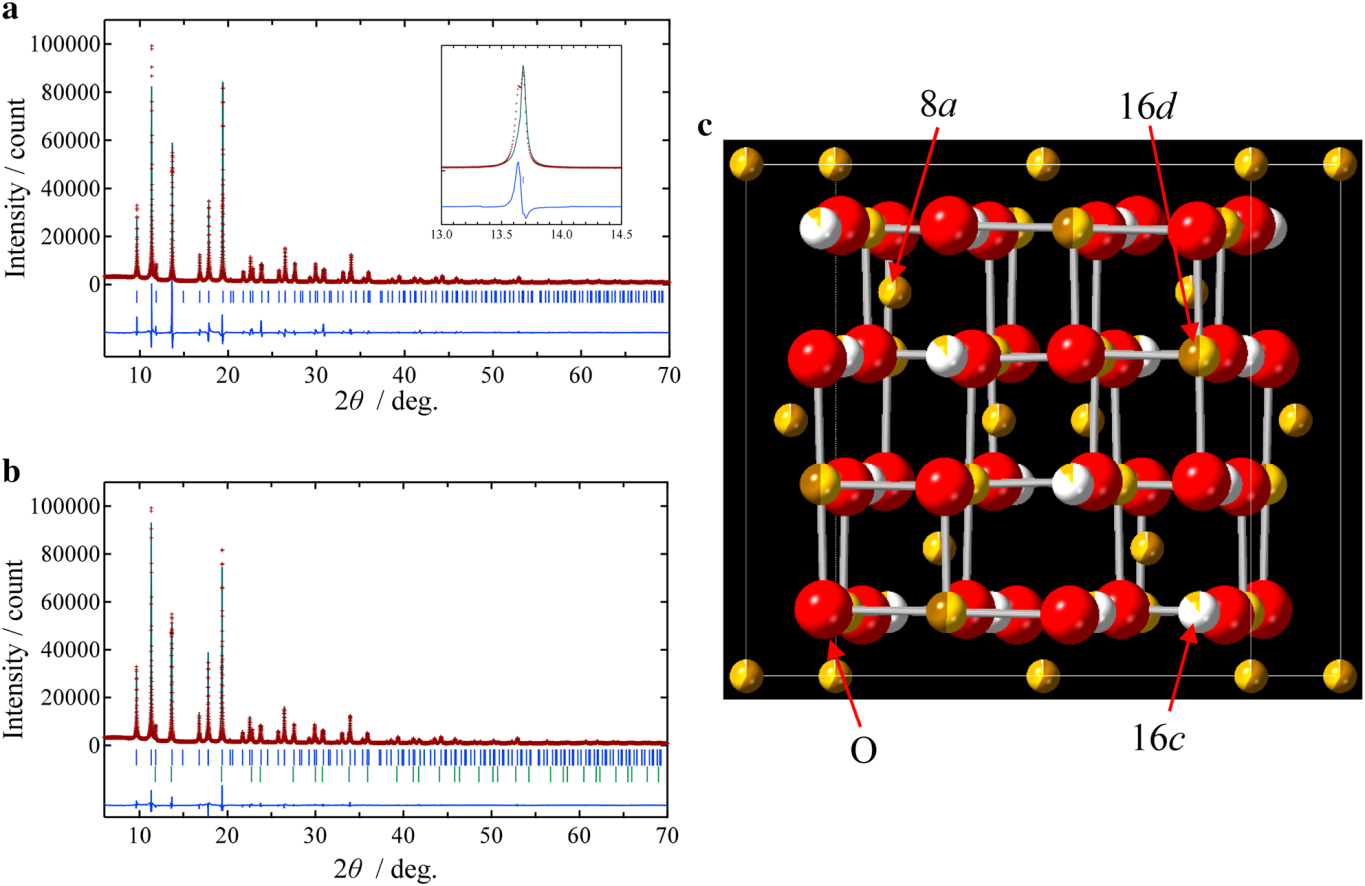
(**a**) Rietveld refinement pattern of Mg–Fe–O under an assumption that the sample is a single phase of the spinel structure. An enlarged image around 13.0–14.5 degrees is also presented as an inset. (**b**) Rietveld refinement pattern of Mg–Fe–O under an assumption that the sample is composed from the Mg-rich spinel oxide and MgO (a rocksalt structure). The red plus marks show observed intensities, and the green solid line represents calculated intensities. The vertical bars indicate positions of allowed Bragg reflections [(**a**) spinel; (**b**) spinel (upper), rocksalt (lower)]. The blue curve at the bottom is a difference between the observed and calculated intensities at the same scale. (**c**) Refined crystal structure of the Mg-rich spinel oxide (Mg_1.651_Fe_1.566_O_4_). This figure was created using the CrystalMaker 10.7.

Since the Mg insertion/deinsertion process must be related to the atomic configuration, we investigated a crystal structure of the Mg-rich iron oxide in detail by a Rietveld refinement using a synchrotron X-ray diffraction pattern. According to the result of preliminary XRD measurement (Fig. 1), ICP-AES (Table 1), and XANES spectra (Fig. 2), the sample can be regarded as Mg_2.446_Fe_1.036_O_4_ with the spinel structure (S. G.: *Fd*-3* m*). Since a total amount of cations is beyond 3 in this formula, the excess amount of the metals should be located at the interstitial site (the 16*c* site) in the spinel structure. Under the assumption, the Rietveld refinement using the synchrotron X-ray diffraction pattern was performed firstly. In the refinement, site occupancies of Mg and Fe were refined to optimize distribution of the metals while keeping the atomic ratio of Mg:Fe:O = 2.446:1.036:4. As a result, the Fe occupancy at the 16*c* site became an inappropriate negative value, and thus we fixed the Fe occupancy as 0. The refinement result is presented in Fig. 4a. It is found that most of Bragg peaks can be fitted well by using a single-phase model of the spinel structure. As can be seen in the enlarged view (2*θ* = 13.0–14.5 degrees), however, there is an extra peak beside that of the spinel phase. Since the extra peaks were supposed to be attributed to MgO (an impurity phase), we performed the Rietveld refinement again under assumption that MgO in addition to the spinel phase was formed due to the high Mg/Fe content. Figure 4b, c, and Table 2 show the refinement pattern, the refined crystal structure of the spinel phase, and the structural parameters, respectively. In the analysis, we decreased a Mg content in the spinel phase taking the MgO amount into account. From the structural parameters (Table 2), it is demonstrated that a revised composition of the spinel structure can be expressed as Mg_1.651_Fe_1.566_O_4_ (theoretical capacity for Mg insertion: 219 mA h g^–1^) and a significant amount of Mg occupies the 16*c* site in the spinel structure. As is well known, if all cations exist at the 16*c* and 16*d* sites (octahedral sites) in the spinel structure, this structure is almost equal to the rocksalt structure (Fig. S1). Considering this relation between the spinel and rocksalt structures, the crystal structure of the Mg-rich Mg_1.651_Fe_1.566_O_4_ can be regarded as an intermediate structure between the spinel and the rocksalt structures.

**Table 2:** Refined structural parameters of Mg-rich Mg–Fe–O under an assumption that the sample is composed from (**a**) the Mg-rich spinel oxide (S. G.: *Fd*-3* m*) and (**b**) MgO with a rocksalt structure (S. G.: *Fm*-3* m*).

a						
Atom	Site	*x*	*y*	*z*	*B*/Å^2^	Site occupancy
Mg1	8*a*	0	0	0	0.25(1)	0.405
Fe1	8*a*	= *x*(Mg1)	= *y*(Mg1)	= *z*(Mg1)	= *B*(Mg1)	0.577
Mg2	16*d*	5/8	5/8	5/8	0.33(1)	0.506(2)
Fe2	16*d*	= *x*(Mg2)	= *y*(Mg2)	= *z*(Mg2)	= *B*(Mg2)	0.495(2)
Mg3	16*c*	1/8	1/8	1/8	0.3(2)	0.118(4)
O1	32*e*	0.3804(1)	= *x*(O1)	= *x*(O1)	0.56(2)	1

b						
Atom	Site	*x*	*y*	*z*	*B*/Å^2^	Site occupancy
Mg4	4*a*	0	0	0	0.21(3)	1
O2	4*b*	1/2	1/2	1/2	0.35(4)	1

The spinel phase can be expressed as Mg_1.651_Fe_1.566_O_4_, and the weight ratio is 69.6%. In this model, the 16*c* site is occupied partially by the excess Mg.

Lattice parameters: *a* = 8.39483(8) Å (spinel) and *a* = 4.2127(1) Å (rocksalt).

*R* factors are *R*_wp_ = 5.69%, *R*_p_ = 4.44%, and *R*_e_ = 2.22%.

**Figure 5:**
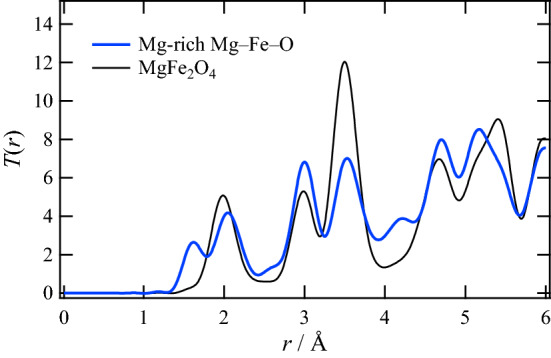
Total correlation function, *T*(*r*), of Mg-rich Mg–Fe–O. That of MgFe_2_O_4_ with the spinel structure is also presented as a reference.

To confirm the existence of excess Mg at the interstitial space (the 16*c* site) from the view point of local structure, a total correlation function, *T*(*r*), of the Mg-rich sample was obtained from an X-ray total scattering measurement, and the function is presented in Fig. 5. As a reference, *T*(*r*) of MgFe_2_O_4_ without excess Mg is also given in this figure. As can be seen, there is a peak at a shorter distance than 2 Å in the Mg-rich Mg–Fe–O whereas this peak does not exist in MgFe_2_O_4_. This difference suggests that there are cations at interstitial positions of the spinel structure in the Mg-rich sample, since interatomic distances in the secondary phase (MgO) should be longer than 2 Å based on the refined lattice constant (Table 2). This result supports the above conclusion that the Mg-rich Mg–Fe–O has the intermediate structure between the spinel and the rocksalt structures.

According to previous works^4^, the Mg insertion into the spinel structure during a discharging process accompanies a phase transition to the rocksalt structure, and this phase transition has a negative influence on cycle performances of spinel-type positive-electrode materials. To overcome this problem, previous works tried to suppress the phase transition of the spinel structure to the rocksalt structure as far as possible. On the other hand, we succeeded here in achievement of the good positive-electrode property by synthesizing the Mg-rich Mg–Fe–O with the intermediate structure of the spinel and the rocksalt structures. This can be regarded as a new strategy to improve positive-electrode properties of MRB.

## Conclusions

In this work, the Mg–Fe–O and Mg–Ni–O samples with the high Mg contents were prepared, and the positive-electrode properties as MRB and the crystal structures were investigated. It is found from the preliminary XRD measurement that the iron-based oxide has the spinel structure whereas the nickel-based oxide has the rocksalt structure. Although the charge/discharge capacity of the Mg–Ni–O sample is poor, the Mg–Fe–O with the Mg-rich composition exhibits good cycle performance. To study the crystal structure of the Mg–Fe–O in detail, the Rietveld refinement is carried out. As a result, it is indicated that the excess Mg occupies the vacant octahedral site in the spinel structure, and the atomic configuration can be regarded as the intermediate structure of the spinel and rocksalt structures. Considering the discharge/charge mechanism of the spinel-type electrode material, it can be concluded that the excess Mg in the Mg-rich Mg–Fe–O has an important role for the good cycle performance.

## Methods

To synthesize the spinel-type Mg–Fe–O with a Mg-rich composition, a Mg-Fe LDH was prepared as a first step, according to literature^27,28^. MgCl_2_·6H_2_O and FeCl_2_·6H_2_O were dissolved into pure water to achieve [Mg]:[Fe] of 2:1, and pH of the aqueous solution was controlled at 13.3 by adding 2.5 mol dm^–3^ KOH aqueous solution. After continuous stirring for 24 h, the obtained precipitate (Mg–Fe LDH) was dried in air at 100 °C for 12 h. The Mg–Fe–LDH was fired in air at 550 °C for 10 h as a second step, and then a final product was obtained. Regarding a rocksalt-type Mg–Ni–O, the sample was prepared by a thermal decomposition of precipitate which was obtained in the similar manner to the Mg–Fe–O. In the preparation process, we used MgCl_2_·6H_2_O and NiCl_2_·6H_2_O as starting materials.

Phases of these samples were identified preliminarily by laboratorial X-ray diffraction measurements (Empyrean, PANalytical), and their metal compositions were evaluated by ICP-AES (ICPE-9000, Shimadzu). Transition-metal valences in the samples were estimated by analyzing XANES spectra measured at BL14B2 (SPring-8). To investigate the crystal structure in detail, synchrotron X-ray diffraction profiles (Bragg profiles) were measured with a wave length of 0.5 Å (BL19B2, SPring-8), and the crystal structure were analyzed by a Rietveld refinement with a Rietan-FP program^29^. X-ray total scattering measurements were also carried out with a wave length of 0.2 Å (BL04B2, SPring-8) to discuss the atomic configuration from the viewpoint of local structure. The scattering data were normalized into Faber-Ziman structure factors, *S*(*Q*), and then transformed to total correlation function, *T*(*r*), by a Fourier transform^30^. This correction process was performed using a standard program^31^.

Positive-electrode properties of the samples were investigated at 90 °C by charge/discharge cycle tests using a three-electrode cell. Each positive electrode was prepared by mixing the electrode material, Super C65, and PTFE with a weight ratio of 5:5:1. The negative and reference electrodes were Mg alloy (AZ31) and Ag, respectively, and the electrolyte was 0.3 mol dm^–3^ [Mg(G4)][TFSA]_2_/[PYR13][TFSA]^32^. The current density was 5 mA g^–1^, and the cut-off potential was − 1.155 ~ 0.845 V versus Ag/Ag^+^ or − 1.6 ~ 1.1 V versus Ag/Ag^+^. These potentials can be converted to those vs. Mg/Mg^2+^, according to literature^32^.

## Supplementary Information

Supplementary Information.

## Data Availability

The datasets used and/or analyzed during the current study available from the corresponding author on reasonable request.
