# Supplementary material for: Positive-electrode properties and crystal structures of Mg-rich transition metal oxides for magnesium rechargeable batteries
Source: Sci Rep. 2022 Oct 27;12:18097. doi: 10.1038/s41598-022-23022-1 (PMC9613765; doi:10.1038/s41598-022-23022-1)
Supplement: Supplementary file 1 — Supplementary Information. [file 41598_2022_23022_MOESM1_ESM.docx]

**Supplementary material**

**Positive-Electrode Properties and Crystal Structures**

**of Mg-Rich Transition Metal Oxides for Magnesium Rechargeable Batteries**

Naoto Kitamura ^1, 2^, Yoichiro Konishi ^1^, Wenli Ma ^1^, Naoya Ishida ^1^,

Toshihiko Mandai ^3^, Chiaki Ishibashi ^1^, Yasushi Idemoto ^1, 2^

^1^ *Department of Pure and Applied Chemistry, Faculty of Science and Technology, Tokyo University of Science, 2641 Yamazaki, Noda, Chiba 278-8510, Japan*

*^2^ Research Group for Advanced Energy Conversion, Research Institute for Science and Technology, Tokyo University of Science, 2641 Yamazaki, Noda, Chiba 278-8510, Japan*

*^3^ Center for Green Research on Energy and Environmental Materials, Center for Advanced Battery Collaboration, National Institute for Materials Science, Tsukuba, Ibaraki 305-0044, Japan*


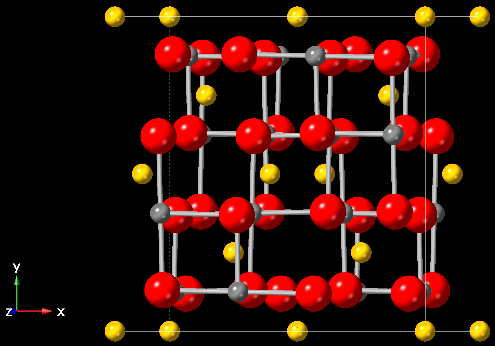


8*a*

16*d*

O


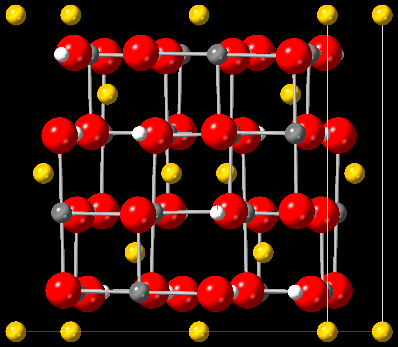


16*c*

**
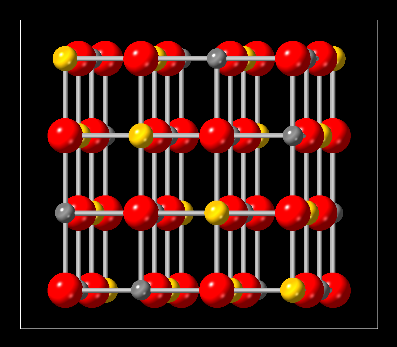
a b c**

Fig. S1 Schematic illustrations of the spinel structure (a), spinel structure with interstitial cations at the 16*c* site (b), and the rocksalt structure (c). In Fig. S1 (c), the yellow and gray spheres are equivalent to the 16*c* and 16*d* sites in the spinel structure, respectively. These figures were created using the CrystalMaker 10.7.
